# Experimental study on the effects of isoflurane with and without remifentanil or dexmedetomidine on heart rate variability before and after nociceptive stimulation at different MAC multiples in cats

**DOI:** 10.1186/s12917-019-2004-8

**Published:** 2019-07-24

**Authors:** Jonathan F. Raue, Mika P. Tarvainen, Sabine B. R. Kästner

**Affiliations:** 1 Small Animal Clinic, University of Veterinary Medicine Hannover, Foundation, Bünteweg 9, 30559 Hannover, Germany; 2 Department of Applied Physics, University of Eastern Finland, Kuopio, Finland; 3 Department of Clinical Physiology and Nuclear Medicine, Kuopio University Hospital, P.O. Box 1627, FI-70211 Kuopio, Finland; 4 Center for Systems Neuroscience Hannover, University of Veterinary Medicine Hannover, Foundation, Bünteweg 9, 30559 Hannover, Germany

**Keywords:** Anaesthesia, Anaesthetic depth, Cats, Dexmedetomidine, Heart rate variability, Isoflurane, Minimum alveolar concentration, Remifentanil

## Abstract

**Background:**

Heart rate variability (HRV) provides information about autonomic nervous system (ANS) activity and is therefore a possible tool with which to assess anaesthetic depth. The aim of the present study was to evaluate the effects of isoflurane, remifentanil and dexmedetomidine on HRV before and after nociceptive stimulation at different anaesthetic depths.

Seven healthy domestic short-hair cats were used, and each cat was anaesthetized three times – group I with isoflurane alone, group IR with isoflurane and a constant rate infusion (CRI) of remifentanil (18 μg/kg/h), and group ID with isoflurane and a CRI of dexmedetomidine (3 μg/kg/h). Minimum alveolar concentration (MAC) values were determined via electrical supramaximal nociceptive stimulation for each treatment group. Nociceptive stimulation was repeated at 3 different MAC multiples (0.75, 1.0 and 1.5 MAC), and electrocardiographic recordings were performed for 3 min before and after stimulation. Only the 1 min epochs were used for further statistical analysis. Electrocardiographic data were exported for offline HRV analysis.

**Results:**

The mean isoflurane MAC ± standard deviation (SD) was 1.83 ± 0.22 vol% in group I, 1.65 ± 0.13 vol% in group IR and 0.82 ± 0.20 vol% in group ID. Nociception was indicated by several HRV parameters, however, with high variability between treatments. The best correlation with MAC was found for the SD of heart rate (STD HR) in group I (*r*_***s***_ = − 0.76, *p* = 0.0001, *r*^*2*^ = 0.46). STD HR was also able to distinguish 0.75 MAC from 1.5 MAC and 1.0 MAC from 1.5 MAC in group I, as well as 0.75 MAC from 1.5 MAC in group ID.

**Conclusions:**

The choice of anaesthetic protocol influences the HRV parameters in cats. Frequency domain parameters respond to nociception at lower MAC levels. The STD HR has the potential to provide additional information for the assessment of anaesthetic depth in isoflurane-anaesthetized cats. The utility of HRV analysis for the assessment of anaesthetic depth in cats is still questionable.

**Electronic supplementary material:**

The online version of this article (10.1186/s12917-019-2004-8) contains supplementary material, which is available to authorized users.

## Background

Measurement of heart rate variability (HRV) provides information in terms of evaluation of the autonomic nervous system (ANS) and the balance of sympathetic and parasympathetic activity [1, 2]. HRV can be analysed by time and frequency domain parameters. Time domain parameters such as STD HR, the root mean square of successive RR interval differences (RMSSD) and the percentage of successive RR intervals that differ by more than 50 ms (pNN50) provide information about beat-to-beat HRV, whereas frequency domain parameters such as high frequency (HF), low frequency (LF) and very low frequency (VLF) are able to show the power distributions of different frequency ranges as they are derived from spectral analysis [3]. For example, a relation of changes in the HF domain to respiratory sinus arrhythmia and, therefore, to parasympathetic tone has been shown. In contrast, changes in the LF domain are connected to sympathetic activity or the activation of both systems [4]. Therefore, the LF/HF ratio is important for the interpretation of the sympathovagal balance.

General anaesthesia and nociceptive stimuli have a significant influence on the sympathovagal balance and, as a consequence, on HRV parameters. Research on the utility of HRV analysis for the assessment of anaesthetic depth has been performed in humans and dogs [5–8]. A dedicated HRV monitoring device has been validated in several species, including dogs [9]. HRV analysis has been studied in awake [10] or decerebrate [11] feline patients, as well as in some anaesthetic and surgical situations [12, 13] but, to the authors’ knowledge, not in cats undergoing different anaesthesia protocols. Therefore, the aim of this study was to evaluate the changes in HRV in cats at different MAC-defined anaesthetic levels with and without nociceptive stimulation using three different anaesthetic protocols. Our hypothesis was that HRV parameters change with increasing isoflurane MAC multiples and nociception and that the choice of additional drugs influences the response.

## Results

The mean duration of anaesthesia was 321 ± SD 35 min in group I, 323 ± SD 29 min in group IR and 367 ± SD 29 min in group ID.

One cat was excluded from group ID due to the development of ongoing 2nd degree atrioventricular blocks after the administration of dexmedetomidine. The blocks disappeared after stopping the CRI of dexmedetomidine. The same cat did not show any signs of arrhythmia in group I or IR. No arrhythmias were observed in the recordings of the other 6 cats in any group. The range of the respiratory rate during artificial ventilation was 11–20/min, with higher values at lower MAC levels where adjustments up to 46/min were needed due to spontaneous breathing attempts.

Isoflurane 1.0 MAC values were 1.83 vol% in group I, 1.65 vol% in group IR and 0.82 vol% in group ID, corresponding to a significant MAC-sparing effect of 55.2% (*p* = 0.003) in group ID but no significant MAC-sparing effect of 9.8% in group IR. In 2 cats, a higher MAC value was observed in group IR than in group I. The prestimulation values of the 3 aforementioned epoch lengths did not differ, therefore only the 1-min epochs were used in further statistical analyses.

**Fig. 1:**
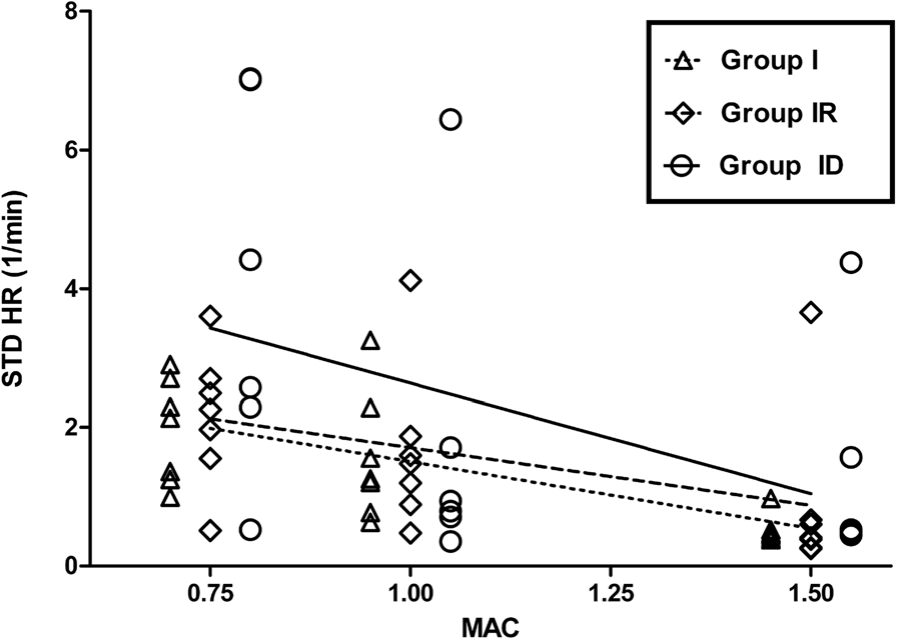
STD HR correlation with MAC. STD HR values (1/min) of 7 cats determined with isoflurane alone (group I, triangles), with isoflurane and a constant rate infusion of remifentanil (18 μg/kg/h IV; group IR, diamonds) and with isoflurane and a constant rate infusion of dexmedetomidine (3 μg/kg/h IV; group ID, circles) at different MAC levels (0.75, 1.0 and 1.5 MAC). Prestimulation values were used for correlation analysis. The correlation coefficients for STD HR were − 0.76 (*p* = < 0.0001) in group I, − 0.56 in group IR (*p* = 0.0085) and in group ID (*p* = 0.0142) with increasing MAC level. The slopes of the best-fit linear regression lines were − 1.932 (*r*^*2*^ = 0.4644, *p* = 0.0007) in group I, − 1.669 (*r*^*2*^ = 0.1975, *p* = 0.0435) in group IR and − 3.189 (*r*^*2*^ = 0.181, *p* = 0.0784; not significant) in group ID

As shown in Fig. 1, the best overall correlation with MAC was found for STD HR in group I (*r* = − 0.76, *p* = < 0.0001), followed by group IR (*r* = − 0.56, *p* = 0.0085) and ID (*r* = − 0.56, *p* = 0.0148). Moderate correlation with MAC was found for HF (ms^2^) in group ID (*r* = − 0.54, *p* = 0.02), LF (ms^2^) in group ID (*r* = 0.48, *p* = 0.04), mean HR in group I (*r* = 0.61, *p* = 0.004) and in group IR (*r* = 0.60, *p* = 0.004), and STD RR in group I (*r* = − 0.51, *p* = 0.02) and in group ID (*r* = 0.55, *p* = 0.02).

Regarding the prestimulation values, as shown in Additional file 1: Table S1, STD HR was significantly lower at 1.5 MAC than at 1.0 MAC and 0.75 MAC in group I (*p* = 0.0012) and was also significantly lower at 1.5 MAC than at 0.75 MAC in group ID (*p* = 0.012). No significant differences between MAC levels were found for STD HR in group IR. The LF (ms^2^) values were significantly lower at 1.5 MAC than at 0.75 MAC in group I (*p* = 0.049). The HR was significantly lower at 1.5 MAC than at 0.75 MAC in group I (*p* = 0.272) and group IR (*p* = 0.049). The lowest overall HRs were observed in group ID.

At 0.75 MAC, the values of group ID were significantly lower than in group IR for the mean HR (*p* = 0.001), LF (n.u.) (*p* = 0.03) and the LF/HF ratio (*p* = 0.03) and significantly higher than in group IR for STD RR (*p* = 0.002), HF (ms^2^) (*p* = 0.0016), HF (n.u.) (*p* = 0.03) and LF (ms^2^) (*p* = 0.015). Also at 0.75 MAC, the values of group ID were lower than in group I for mean HR (*p* = 0.0014) and higher than in group I for STD RR (*p* = 0.002), HF (ms^2^) (*p* = 0.0016) and LF (ms^2^) (*p* = 0.015). The HF power of group ID was higher than in group IR throughout all MAC levels (Additional file 1: Table S1).

After nociceptive stimulation, STD HR increased significantly in groups I and IR at all MAC multiples. As shown in Additional file 1: Table S1, poststimulation values of several other parameters (especially frequency domain parameters LF n.u. and the LF/HF ratio) were altered by nociceptive stimulation at 0.75 MAC and 1.0 MAC in all treatment groups, but none of them were able to depict the stimulation at all anaesthetic depths. Most of the significant changes after stimulation were observed at 1.0 MAC throughout all groups. The comparison of the individual pre- to poststimulation change values showed only few significant differences between groups and MAC levels, such as the higher difference value of LF (ms2) of group ID compared with group IR at 1.0 MAC, for example, as shown in the table.

## Discussion

In the current study, based on its strong correlation with MAC, STD HR seemed to be the most useful HRV parameter to indicate insufficient depression of nociception or insufficient anesthetic depths. However, HRV is significantly influenced by drug choice, thereby limiting the utility of standard HRV parameters across different anaesthetic protocols.

### MAC

The MAC concept is a standard method for evaluating the efficiency of inhalant anaesthetics [14], enabling the results to be reproducible and comparable. One MAC is defined as the end-tidal concentration of an anaesthetic agent that prevents gross muscular movement in response to a painful stimulus [15]. Several stimulation techniques exist for MAC determination, especially in veterinary anaesthesia, including the tail clamping method and electrical stimulation protocols. Both techniques have been validated and compared in dogs and rabbits, with similar results [16], but to our knowledge, these techniques have not been validated in cats. The constant voltage electrical stimulation protocol as described for dogs was used in our study after confirmation in pre-trials that maximal stimulation was achieved in cats as well. The individual MAC values of the cats in group I with a mean MAC of 1.83 vol%were close to those reported in the literature [17]. This implies comparability of the stimulation method.

Remifentanil is a potent opioid with a short mean half-life of 15.7 min in cats [18]. Due to its pharmacological features, including fast elimination via extrahepatic metabolism, a cumulative effect is unlikely. Studies concerning the isoflurane-sparing effect of remifentanil in cats show some variation in their results [19]. Generally, the effect seems to not be as high as that in other species, such as rats [20] and dogs [21]. One study did not reveal any MAC-sparing effect from several remifentanil CRI dosages in cats undergoing isoflurane anaesthesia [22], whereas other studies found a relatively constant MAC-sparing effect between 23 and 30% [23] or a reduction of 15.6% [24]. In our study, the MAC in group IR was slightly reduced (9.8%), but there was a large variation among individuals, resulting in the lack of statistical significance. The aforementioned MAC increase in two cats in group IR can be explained by the central stimulating effects of opioids in cats [19]. The dosage of 18 μg/kg/h was chosen in accordance with clinical experience as well as published doses. Previous experiments with cats did not reveal any beneficial effects on isoflurane requirements at higher CRI dosages, implying a possible ceiling effect [24]. Several studies have been performed to describe pharmacological variables as well as clinically useful dosages. Because these studies had different goals, their proposed CRI rates are not comparable. One study stated a constant infusion rate of 42 μg/kg/h as the median analgesic effective dosage [22], whereas in another study, a dosage of 13.8 μg/kg/h was sufficient for ovariohysterectomy, and 18 μg/kg/h prevented movement after supramaximal stimulation. However, a possible mismatch between anaesthetic immobility and analgesia must be considered [22]. Dexmedetomidine is an α_2_-adrenoceptor agonist with analgesic and muscle relaxing properties, and both features can influence the MAC. The MAC-sparing effect in group ID was 55.2% in our study. This finding corresponds to the findings from studies on other species. An epidural administration of dexmedetomidine was able to reduce the isoflurane requirements up to 33% in dogs [25], and a CRI of dexmedetomidine in dogs at the same rate as in the present study had a MAC-sparing effect between 41% [26] and 59% [27]. In cats, pharmacokinetic studies have shown a dose-dependent reduction in isoflurane requirements with an estimated maximal sparing effect of approximately 80% [28]. Because these were studies with target-controlled dexmedetomidine infusions [28, 29] or studies that included only a short-term infusion of 2 μg/kg/min over 5 min [30], their results cannot be directly compared to ours.

The equilibration phase in our study (the time between starting the remifentanil or dexmedetomidine CRI and the first noxious stimulus) was set to 60 min. The aforementioned short half-life of remifentanil indicates a time period of approximately 75 min to reach steady state (5 times the half-life) [31]. Therefore, steady state was not necessarily reached at the first stimulus in group IR but was likely achieved by the following stimuli and measurements. In contrast, because the half-life of dexmedetomidine is much longer, at a mean of 198 min [30], a steady state at the beginning was unlikely reached in any of the experiments in group ID. A bolus, on the other hand, might have led to undesired high concentrations. This should be considered a possible source of error in our results for group ID as well as group IR, especially because plasma concentrations of dexmedetomidine and remifentanil were not determined.

Another considerable limiting factor is the calculation of the different MAC levels. The amount of isoflurane was changed from 1.0 MAC to 0.75 MAC or 1.5 MAC, but the CRI remained the same, which might have led to different anaesthetic planes when comparing the groups.

### Epoch lengths

The circumstances during surgical procedures can change rapidly, and anaesthesia often requires spontaneous adjustments. Therefore, if HRV analysis is considered for anaesthetic monitoring, short measurement epochs or time-varying analysis methods are desirable to enable the detection of changes as early as possible [5]. In contrast, the guidelines presented by The Task Force of the European Society of Cardiology and the North American Society of Pacing and Electrophysiology [3] recommend longer intervals for adequate HRV analysis, including a minimum of 1 min for HF and 2 min for LF analysis, as well as a general recommendation of 5-min intervals. Newer studies did not show disadvantages in the use of even shorter epochs in the evaluation of some time-domain parameters such as SDNN [32, 33]. Two-minute epochs have been used in a recent study with beagle dogs [26]. In particular, cats tend to show movement or signs of intraoperative awareness very quickly and, sometimes, unexpectedly. Three epochs with a maximum of 3 minutes were analysed in this study. Because the HRV values of the different prestimulation epochs did not differ, the 1-min epochs were used for further statistical analysis. Additionally, nociceptive stimulation could be detected in only the 1-min poststimulation epoch measurements, which were used for further statistical analysis, while longer measurements usually revealed a re-adjustment to the prestimulation values.

HRV measurements are also affected by CO_2_ and respiratory rate [34]. Both were held in physiological ranges as close as possible. Still, a confounding effect cannot be excluded, especially in these ultra-short-term measurements.

### Drug influences and HRV analysis

Three different anaesthetic, sedative or analgesic drugs were used in this study, each of which influence HRV parameters, as shown in different species [6, 10, 35–37]. Nevertheless, because all groups except group I were treated with combinations of anaesthetics and there was no awake control group, the exact impact of each drug in this study can only be assumed.

The consistent anaesthetic factor throughout all groups in our study was the inhalant anaesthetic isoflurane. Changes in HRV under isoflurane anaesthesia have been described in several studies with humans [6, 7], but few data are available concerning its special influence on HRV in other species. Data from a study with beagle dogs [26] show a dose-dependent effect of isoflurane on HRV parameters, including low HF and high LF values as well as continuously high heart rates. These findings are quite similar to those of the present study and are likely a result of an increase in sympathetic activity, which can occur as a reflex to isoflurane-induced low systemic vascular resistance [38, 39]. In contrast, even more than those in the beagle study, the measured HRs of the cats in group I were reduced at higher MAC levels, which might be explained by a reduction in sympathetic tone at higher isoflurane concentrations [39, 40]. Nevertheless, the examination of conscious cats compared to anaesthetized cats was not part of the present study, and therefore, the effect of isoflurane on HRV analysis cannot completely be quantified.

Generally, there are two ways HRV analysis can be influenced by the addition of drugs other than the given volatile anaesthetic. First, the additive affects the required amount of inhalant for an equipotent effect on the MAC, lowering the influence of the inhalant on HRV. Second, the potency of the additive in changing HRV must also be considered.

Remifentanil is a strong analgesic that acts mainly via μ_1_-agonism [41]. Its effects on cardiovascular variables, but not on HRV parameters, have been previously examined in cats [42]. In the same study, bradycardia and reduced blood pressure were commonly observed side effects. In contrast to that study as well as to the aforementioned beagle study [26], mean HRs in group IR were not significantly reduced compared with those in group I at the corresponding MAC level. Instead, even higher HRs were observed at 0.75 and 1.0 MAC. This observation might be explained by opioid-induced excitatory effects and the following increase in sympathetic tone in cats [23], whereas the lower HRs at 1.5 MAC are likely the result of the relatively increased and now predominant influence of isoflurane. However, as an overall impression, most of the HRV parameter values of group IR in the present study are close to those of group I, which indicates that either there were no pronounced effects of remifentanil on HRV parameters or the effects were similar to those of isoflurane.

In contrast to remifentanil, dexmedetomidine reduced the HR at all MAC levels. Additionally, in agreement with results reported from beagle dogs [26], the highest STD HR values, as well as the lowest LF n.u. and highest HF n.u. values, were found at 0.75 MAC, leading to the lowest LF/HF ratio at all MAC levels. Dexmedetomidine reduces sympathetic tone via central α_2_-adrenoceptor agonism and peripheral α_2β_-adrenoceptor activation, leading to increased vascular resistance and, as a reflex, to a decrease in heart rate [43, 44]. The prominent MAC-sparing effect, which was present in group ID, implies a great presence of a cardiovascular-suppressing α_2_ agonist and therefore explains the constantly low prestimulation HR in group ID throughout all MAC levels. Because minor changes of ET_ISO_ were enough to go from 0.75 MAC to 1.0 or 1.5 MAC, only slightly different anaesthetic depths were achieved; therefore, most HRV parameters failed to discriminate between those levels. For the same reason, nociceptive stimulation could be depicted by HR and other parameters throughout all MAC levels in group ID. However, in contrast to group I and IR, STD HR was not a reliable indicator of nociception at all MAC levels. The most likely explanation for the increase of STD HR after nociception is sympathetic activation, leading to more overall ANS activity.

HRV analysis can be performed by obtaining time domain and frequency domain parameters. Sometime domain parameters, such as the SDANN, SDNNI and HRV Triangular Index, are useful only for longer measurements. For other parameters, such as the SDNN, NN50, pNN50 or RMSSD, shorter epochs of 60–240 s have been studied [2]. SDNN, or STD RR, as named in the Kubios program, is believed to show more reliability in longer time periods up to 24 h. STD RR displays the standard deviation of NN intervals, whereas STD HR shows the standard deviation of the instantaneous HR. The latter does not necessarily show the same changes as STD RR because it is the mean of only very few heartbeats and does not use the exact RR interval time. Nevertheless, it is interesting that in our study, STD HR showed greater correlations. In the frequency domain, the choice of the studied frequency bands (HF, LF, VLF and ULF) also depends on the measured epoch lengths. ULF and VLF are not meaningful in the context of 1 min epochs and have therefore not been examined in the present study, and only the aforementioned shorter-term time domain parameters have been considered for statistical analysis. Both domains are influenced by both branches of the ANS in a complex manner [2], and none of those domains or parameter can be regarded separately.

Currently, there are no defined standards for HRV frequency bandwidth in cats. For frequency domain analysis, the HF, LF and VLF bands of the present study were chosen in accordance with previous HRV studies with cats [10, 45, 46]. In most studies with other species, the HF band is usually set at 0.15–0.4 Hz but can be expanded to lower than 0.15 Hz and up to 1 Hz. The HF band is thought to mainly reflect influences of respiratory sinus arrhythmia. Because cats tend to have slightly higher respiratory rates and according to the rates found in the present study, the HF band was set to 0.15–0.833 Hz (equivalent to a respiratory rate of approximately 10–50/min). These settings have been found to work best for consistent conditions. Despite artificial ventilation, as provided in the present study, most cats showed spontaneous breathing attempts, especially at low MAC levels, after nociceptive stimulation. Irrespective of the interference with our measurements as mentioned above, this limiting factor must be considered for possible anaesthetic monitoring via HRV analysis in clinical settings.

Generally, as seen in Additional file 1: Table S1, there was a large individual overlap, especially in frequency domain parameter values within treatment groups or MAC multiples, as already reported in dogs [26]. Therefore, the ratios of these spectral portions might be more useful for the interpretation and inter-individual comparison of anaesthetic depth than absolute numbers.

Of note, we did not screen the cats via echocardiography before the experiments. Although none of the cats showed any abnormality at the clinical examination, including auscultation and baseline ECGs, it is still possible that underlying heart diseases might have been overlooked.

Few correlations have been found in the present study. The strongest correlation with MAC was found in STD HR. The correlation coefficient value of r = − 0.76 is good, but not as high as expected. The regression analysis results of our study indicate a non-linear relationship of MAC and STD HR. This should be considered as a possible limitation if HRV parameters are used for real time anaesthetic depth monitoring in a clinical setting.

Changes in several HRV parameters within groups were observed with increasing MAC and after nociception, supporting our hypothesis. Nevertheless, these changes were not as big as expected. The comparison between the treatment groups at the same MAC levels resulted in a wide variation of HRV parameters with decreasing MAC, which might be explainable by the preponderance of isoflurane effects at higher MAC levels.

A limitation of this study was, that the number of animals in this orientation study has been chosen in accordance with previous data from dogs [26] and for ethical reasons. The high variability of HRV parameters across the different protocols might have contributed to limited detection of true differences and underpowered results.

## Conclusions

The anaesthetic drug choice influences the HRV parameters in cats. Frequency domain parameters respond to nociception at low MAC levels. The STD HR has the potential to provide additional information to the common monitoring parameters for the assessment of anaesthetic depth in isoflurane-anaesthetized cats. The utility of HRV analysis for the assessment of anaesthetic depth in cats especially in clinical and surgical settings is still questionable because of the great inter-individual overlap of the HRV parameter values and their variable response to different anaesthetic protocols.

## Methods

### Animals

Seven adult experimental European domestic short-hair cats (five male-neutered, two female, one female-spayed) were used in this study. The cats were owned and provided by the Institute for Parasitology (Dept. of Infectious Diseases) of the University of Veterinary Medicine in Hannover, Germany. The mean age ± SD was 5.6 ± 3.0 years, and the mean body weight ± SD was 4.5 ± 0.96 kg. All cats were considered healthy based on a general and neurological physical examination, haematology and blood biochemistry. The animals were fasted for 8 h, and water was offered until 1 h prior to anaesthesia. One animal underwent the test protocol per day, and anaesthesia was started at the same time every day to exclude influences of a circadian rhythm on the measurements. After the experiments, all cats recovered and were brought back to their home facility. None of the cats were euthanized, and rehoming was intended.

The study was performed according to the German animal protection law after review and approval by the ethical committee for animal experimentation of the Federal State Office for Consumer Protection and Food Safety of Lower Saxony, Germany (approval number: 33.12–42502–04-10/0102).

### Experimental design

The present study was performed in an experimental prospective and complete crossover design. Each cat was included in each of 3 experimental treatment groups, which were defined by different anaesthesia protocols. The individual group order for each cat was randomized, and a wash-out period of at least 8 days was given between the experiments. After induction and instrumentation, 1.0 MAC was determined individually by supramaximal nociceptive stimulation. Under the same anaesthesia protocol, 3 different isoflurane MAC multiples were given, and nociceptive stimulation was performed at each level. The measurements were performed at 1.0 MAC first and afterwards at 0.75 MAC and 1.5 MAC in randomized order. One measurement was performed at each MAC level; 0.75 MAC and 1.5 MAC were calculated based on the individual isoflurane 1.0 MAC of each cat. The remifentanil and dexmedetomidine constant rate infusion (CRI) remained unchanged.

While the main investigator was aware of the treatment group, the observers of the cats’ clinical reactions were blinded to the anaesthetic protocol.

### Anaesthesia

Group I received only isoflurane, group IR received isoflurane and a CRI of remifentanil (18 μg/kg/h IV), and group ID received isoflurane and a CRI of dexmedetomidine (3 μg/kg/h IV). Isoflurane in all groups was administered in 100% oxygen. Remifentanil and dexmedetomidine were diluted in saline solution (0.9% NaCl) in a ratio that allowed setting the CRI at a rate of 5 ml/kg/h in both groups. Group I received a CRI of saline solution without additive at the same infusion rate.

### Instrumentation

On the day of the experiment, an intravenous catheter was placed in a cephalic or saphenous vein. Induction of anaesthesia was performed by administration of 5 vol% isoflurane in 100% oxygen (flow rate 5 L/min) in an induction chamber until loss of the righting reflex, followed by mask induction until endotracheal intubation was possible. The cats were placed in right lateral recumbency and connected to a circle breathing system. The CRI was started, and the isoflurane level was set to a value slightly above the estimated 1.0 MAC for each group. An equilibration phase of 60 min began at the start of the CRI. Isoflurane and CO_2_ were measured via infrared spectroscopy with a multiparameter anaesthesia monitor.^1^ The monitor was calibrated with a reference gas mixture (5.00% CO_2_, 33.0% N_2_O, 2% desflurane and N_2_ as balance gas) on the same day prior to each experiment. SpO_2_ was measured via pulse oximetry with the same monitor. Eucapnia (respiratory rate 10–20/min, tidal volume 10–20 ml/kg, 0 PEEP; 35–45 mmHg end-tidal CO_2_) was provided by artificial ventilation. To reduce possible contamination of the end-expired gas samples by inspired gas, the samples were obtained via a needle that had been inserted into the endotracheal tube directly rostral of the epiglottis. The body temperature was measured with an oesophageal probe and kept in physiological ranges (36.7–38.9 °C) with a warm air blanket. Blood pressure measurements were performed non-invasively via the Doppler technique with a sensor placed on the craniomedial aspect of the metatarsus or ventral aspect of the tail, distal to the cuff of the manometer [47]. Measurements were performed at each MAC level to detect and prevent severe hypotension (MAP < 60 mmHg). The ECG measurements were performed with a Televet 100 monitoring system.^2^ The four ECG surface electrodes were placed palmar and plantar on the paws or, if the signal was too low, laterally on both sides of the chest. The connected Televet ECG monitor transmitted the signal to a corresponding personal computer via Bluetooth for recording and R peak detection with Televet Software.^3^

The nociceptive stimulation was performed after connecting a square pulse stimulator^4^ (settings: 50 V, 50 Hz and 10 ms) to 2 stimulation electrodes, which were placed subcutaneously in the middle third of the right medial ulnar region, approximately 4–5 cm apart from each other.

Additionally, 3 electroencephalogram electrodes were placed subcutaneously on the head for data collection in another study.

After each experiment, anaesthesia was discontinued, and all electrodes and catheters were removed from the cat; a single dose of meloxicam (0.1 mg/kg) was given subcutaneously to prevent possible post-experimental pain or inflammatory reactions in the stimulation area.

### MAC determination

MAC was determined individually after each instrumentation period. A standardized supramaximal stimulation protocol [16], including two single stimuli and two continuous stimuli of 3 s, with pauses of 5 s, was applied. The protocol was stopped before finishing all four stimuli if a positive reaction was already observed. Gross movement of the head, the legs (excluding the stimulated leg) or the tail was defined as a positive reaction, whereas swallowing, tongue or ear movement, eye movement, spontaneous breathing or chewing were defined as negative reactions. Due to the results of the preparation phase of this study, a one-minute time period after stimulation was allowed to show a positive reaction, because some cats tended to exhibit a delayed reaction.

A 20-minute equilibration phase without changing the anaesthesia settings was allowed after each change in vaporizer settings before stimulation. Using the bracketing method [48], the ET_ISO_ level was lowered or raised by 0.2 vol% depending on the observation of a positive or negative reaction, followed by another equilibration phase after reaching the desired ET_ISO_ level. Finally, the ET_ISO_ level was lowered or raised by 0.1 vol%, and the individual 1.0 MAC was defined as the arithmetic mean of those two ET_ISO_ values that prevented respectively permitted a positive reaction after supramaximal nociceptive stimulation.

### HRV analysis

The ECG recordings were checked visually for the incidence of arrhythmia. The R peaks were detected automatically during offline analysis with the Televet software. In the case of false detection, manual correction was performed. The RR interval data were exported and further analysed with an HRV analysis programme^5^ [49].

VLF trend components (frequencies below 0.025 Hz) were removed using a smoothness priors method [50]. The RR data were interpolated at 4 Hz to obtain equidistantly sampled data, and the RR power spectrum was estimated using an autoregressive (AR) model of order 16. HRV was then assessed by computing time domain parameters such as the mean heart rate (HR), standard deviation (SD) of the HR (STD HR), standard deviation of NN intervals (SDNN) and percentage of successive NN intervals that differ by more than 50 ms (pNN50). Other time domain parameters have not been further processed due to the epoch length (see discussion section). In the frequency domain, the power of low frequency and high frequency bands was computed both in absolute and normal units (n.u., absolute power of a frequency band divided by the summed absolute power of the LF and HF bands), and the power ratio LF/HF was computed. The frequency bands were defined as LF at 0.025–0.15 Hz and HF at 0.15–0.833 Hz [10, 45]. Analysis was performed on 3 epochs with a length of 1, 2 and 3 min before and after stimulation, whereby all poststimulation epochs started directly after the end of stimulation. Only the 1-min epochs were used in further analysis.

### Statistical analysis

For statistical analysis, commercial software^6^ was used. The HRV parameters were tested for normal distribution with the Kolmogorov-Smirnov test. The non-parametric Wilcoxon signed-rank test for repeated measures was used to compare pre- and poststimulation values. The individual difference between pre- and poststimulation values was calculated. The Friedman test and post hoc tests (Dunn’s multiple comparison test) were performed to compare prestimulation values and the pre−/poststimulation difference of different MAC levels within a group. Due to missing data of one cat in group ID, the Skillings-Mack test was used to compare prestimulation values and the pre−/poststimulation difference between the treatment groups at the same MAC instead of the Friedman test, followed by Wilcoxon signed rank tests and Bonferroni type I error correction in case of statistical significance. To analyse the correlation of HRV parameters to MAC values, Spearman’s rank correlation and linear regression analysis were used. The significance level was defined as *p* < 0.05.

## Additional file

Additional file 1: **Table S1.** HRV parameter values before and after nociceptive stimulation. Values of selected HRV parameters and their differencesdetermined at 1-minute intervals before and after nociceptive stimulation in 7 European domestic short-haircats (6 in group ID) during anaesthesia with isoflurane alone (I), with isoflurane and remifentanil (IR, 18 μg/kg/h IV) or with dexmedetomidine (ID, 3 μg/kg/h IV) at different MAC-defined anaesthetic depths. Data are reported as the median (minimum; maximum). (XLSM 14 kb)

## Data Availability

The datasets used and analysed in the current study are available from the corresponding author on reasonable request.

## Abbreviations

ANS: Autonomic nervous system
AR: Autoregressive
CRI: Constant rate infusion
ECG: Electrocardiogram/graph
ET_ISO_: End-tidal concentration of isoflurane
FFT: Fast Fourier transform
HF: High frequency
HR: Heart rate
HRV: Heart rate variability
I: Isoflurane alone
ID: Isoflurane and a constant rate infusion of dexmedetomidine
IR: Isoflurane and a constant rate infusion of remifentanil
LF: Low frequency
MAC: Minimum alveolar concentration
nu: normalized units
p50NN: percentage of successive RR intervals that differ by more than 50 ms
RMSSD: Root mean square of successive RR interval differences
RR interval: Interval between two consecutive Rpeaks in the electrocardiogram
SD: Standard deviation
SDANN: Standard deviation of the average NN intervals for each 5 min segment of a 24-h HRV recording
SDNN: Standard deviation of NN intervals
STD HR: Standard deviation of heart rate
Triangular Index: Integral of the density of the RR interval histogram divided by its height
VLF: Very low frequency

## Footnotes

1 Datex Ohmeda Compact Monitor, GE Healthcare, Fairfield, Connecticut, USA

2 Televet 100, provided by Rösch & Associates Information Engineering GmbH, Frankfurt am Main, Germany

3 Televet 100, version 4.2.0, provided by Rösch & Associates Information Engineering GmbH, Frankfurt am Main, Germany

4 Grass S48 Square Pulse Stimulator, Astro-Med, West Warwick, Rhode Island, USA

5 Kubios HRV, version 2.0, Biosignal Analysis and Medical Imaging Group, Department of Physics, University of Kuopio, Kuopio, Finland

6 GraphPad Prism, version 5.01, GraphPad Software, La Jolla, California, USA
